# Positive impact of a clinical goal-directed protocol on reducing cardiac arrests during potential brain-dead donor maintenance

**DOI:** 10.1186/s13054-016-1484-1

**Published:** 2016-10-11

**Authors:** Glauco A. Westphal, Elisabeth Coll, Rafael L. de Souza, Silvana Wagner, Artur Montemezzo, Fernanda Carolina Cani de Souza, Gabriel Torres, Stefan Halla, Tiago C. Carnin, Míriam C. Machado, Eduardo Berbigier, Fernando Busetto, Ivonei Bittencourt, Karine Gerent, Bruno S. de Souza, Manoel Tassinari, Joel de Andrade

**Affiliations:** 1 Central de Transplantes do Estado de Santa Catarina, Florianópolis, Santa Catarina Brazil; 2 Universidade da Região de Joinville, Joinville, Santa Catarina Brazil; 3 Organización Nacional de Trasplantes, Madrid, Spain; 4 Centro Hospitalar Unimed de Joinville, Joinville, Santa Catarina Brazil; 5 Hospital Regional Homero de Miranda Gomes, São José, Santa Catarina Brazil; 6 Hospital São Francisco, Concórdia, Santa Catarina Brazil; 7 Hospital Municipal São José, Joinville, Santa Catarina Brazil; 8 Hospital Santa Isabel, Blumenau, Santa Catarina Brazil; 9 Hospital Marieta Konder Bornhausen, Itajaí, Santa Catarina Brazil; 10 Hospital e Maternidade São José, Jaraguá do Sul, Santa Catarina Brazil; 11 Rua Doutor Plácido Gomes, 500 Bairro Anita Garibaldi, 89.202-050 Joinville, Santa Catarina Brazil

**Keywords:** Organ donor, Organ transplantation, Donor management, Donor management goals, Checklist

## Abstract

**Background:**

The disproportion between the large organ demand and the low number of transplantations performed represents a serious public health problem worldwide. Reducing the loss of transplantable organs from deceased potential donors as a function of cardiac arrest (CA) may contribute to an increase in organ donations. Our purpose was to test the hypothesis that a goal-directed protocol to guide the management of deceased donors may reduce the losses of potential brain-dead donors (PBDDs) due to CA.

**Methods:**

The quality improvement project included 27 hospitals that reported deceased donors prospectively to the Transplant Center of the State of Santa Catarina, Brazil. All deceased donors reported prospectively between May 2012 and April 2014 were analyzed. Hospitals were encouraged to use the VIP approach checklist during the management of PBDDs. The checklist was composed of the following goals: protocol duration 12–24 hours, temperature > 35 °C, mean arterial pressure ≥ 65 mmHg, diuresis 1–4 ml/kg/h, corticosteroids, vasopressin, tidal volume 6–8 ml/kg, positive end-expiratory pressure 8–10 cmH_2_O, sodium < 150 mEq/L, and glycemia < 180 mg/dl. A logistic regression model was used to identify predictors of CA.

**Results:**

There were 726 PBDD notifications, of which 324 (44.6) were actual donors, 141 (19.4 %) CAs, 226 (31.1 %) family refusals, and 35 (4.8 %) contraindications. Factors associated with CA reduction included use of the checklist (odds ratio (OR) 0.43, *p* < 0.001), maintenance performed inside the ICU (OR 0.49, *p* = 0.013), and vasopressin administration (OR 0.56, *p* = 0.04). More than three interventions had association with less CAs (OR 0.19, *p* < 0.001). After 24 months, CAs decreased from 27.3 % to 14.6 % (*p* = 0.002), reaching 12.1 % in the following two 4-month periods (*p* < 0.001). Simultaneous increases in organ recovered per donor and in actual donors were observed.

**Conclusions:**

A quality improvement program based on education and the use of a goal checklist for the management of potential donors inside the ICU is strongly associated with a decrease in donor losses and an increase in organs recovered per donor.

**Electronic supplementary material:**

The online version of this article (doi:10.1186/s13054-016-1484-1) contains supplementary material, which is available to authorized users.

## Background

The most realistic option to mitigate the imbalance between the high demand for organ transplantations and the low number of transplantations performed is to maximize the use of organs from brain death (BD) donors [1]. This maximization depends on reducing the underreporting of BD, family refusals, incorrect contraindications, and potential brain-dead donor (PBDD) loss after cardiac arrest (CA) [1–3].

Many donors are lost because they are not properly managed during the first 24 hours [1]. This shortfall highlights the importance of the proactive contribution of intensive care professionals to mitigate the imbalance between supply and demand of organs for transplantation [1, 4].

In 2011, a joint action of the Brazilian Association of Critical Care Medicine and the Brazilian Association of Organ Transplantation resulted in the preparation of the Brazilian Guidelines for Potential Multiple Organ Donors. The initiative aimed to provide parameters of standard care for PBDDs and to reduce loss of donors because of management failures [5, 6]. Guidelines usually fail to achieve rapid impacts on bedside manner changes and rarely consider their practical applicability [7], and the large-scale transformation of the best scientific evidence into clinical practice is a challenge that may take years [5]. The establishment of protocols may help reduce this time, and the management of the process guided by checklists may play a key role in enabling “real-time route corrections” [8]. The Surviving Sepsis Campaign experience is a practical example of the large-scale adoption of a multifaceted model driven by treatment goals [7]. Its positive effects on outcomes result from organizational adjustments which could be adapted to the context of potential organ donor management without requiring additional resources.

Meeting care goals in PBDD management designed to restore the respiratory, cardiovascular, and endocrine-metabolic physiology during the period preceding organ harvesting is associated with an increased number of organs transplanted per donor [1, 9–13] and reduced loss of donors by CA [1, 14].

In a pilot study we reported the association between the managed BD protocol guided by a goal checklist and the decrease in CAs among PBDDs [14]. Recognizing both the challenge and the importance of reproducing those results on a large scale, we conducted a multicenter program to improve the standard of care for PBDDs. The BD protocols were guided by an adaptation of the classical “VIP” approach, a mnemonic method proposed by Max Weil and Herbert Shubin to systematize and simplify a sequence of fundamental principles for the management of shock as “Ventilation, Infusion, and Pumping”. The post scriptum “PS” has been added to refer to “Pharmacological support and some Specificities”, composing the acronym VIPPS [15, 16].

Our purpose is to describe a quality improvement project that includes the implementation of a checklist based on the VIP approach for the management of potential multiple organ donors and to analyze its impact on the occurrence of CA.

## Methods

### Study design

This is a quality improvement project with prospective data collection in 27 hospitals that reported deceased donors to the Transplant Center of the State of Santa Catarina, Brazil, over six 4-month periods from May 2012 to April 2014. The hospitals were encouraged to use a goal checklist for the management of PBDDs.

### Design of the goal checklist for case management

The main recommendations from the Brazilian Guidelines for Potential Multiple Organ Donors [5, 6] were pooled to create a goal checklist based on the adapted form of the VIP approach (Additional file 1) [15, 16], to achieve nine goals to be met upon protocol completion: (1) adequate mechanical ventilation (tidal volume ranging from 6 to 8 ml/kg predicted weight + positive end-expiratory pressure (PEEP) ranging from 8 to 10 cmH_2_O + plateau pressure < 30 cmH_2_O) and FiO_2_ titration to obtain SaO_2_ > 90 %; (2) mean arterial pressure (MAP) ≥ 65 mmHg; (3) diuresis ranging from 1 to 4 ml/kg/h; (4) temperature ≥ 35 °C upon protocol completion; (5) vasopressin when a vasoconstrictor is required; (6) corticosteroids; (7) blood glucose < 180 mg/dl; (8) serum sodium < 150 mEq/L; and (9) protocol duration ranging from 12 to 24 hours.

The compliance with the items was supervised by inhospital transplant coordinators, who alerted the care team if any noncompliance was observed.

### Hospital selection

All hospitals that reported deceased donors to the Transplantation Center were selected. The eight hospitals with the highest numbers of BD notifications (60 % of actual organ donations) were selected to perform onsite training of inhospital transplant coordinators and ICU teams. The training of teams from the other hospitals was restricted to biannual meetings of transplant coordinators. The hospitals were classified according to characteristics that may affect performance.

### Educational material and implementation and training meetings

The main recommendations from the Guidelines, the management checklist and case management instructions, were introduced to all inhospital transplant coordinators of the state in April 2012. Extensive material on the Guidelines and the checklist was provided on a website. A brief version of the Guidelines was handed out at face-to-face meetings with the inhospital transplant coordinators.

A training team consisting of intensivists and nurses conducted onsite training in the hospitals with the best notification and donation performances. Intensivists and nurses were available for consultation by telephone to the care teams.

Partial data on institutional and state transplant system performance were disclosed and discussed every 6 months during the regular meetings of the inhospital transplant coordinators.

### Study population

All patients diagnosed with BD, older than 14 years of age, prospectively and consecutively notified during the observation period were included. The diagnosis of BD was established pursuant to Resolution No. 1480/97 of the Federal Council of Medicine. Additionally, patients were characterized as PBDDs at the time that the diagnostic investigation for BD was started [17]. Given that the maintenance protocol is always stopped in cases of family refusal or clinical contraindication for organ donation after BD confirmation, cases of family decline and clinical contraindications were excluded from the analysis. Cases with insufficient information to conduct the data analysis were also excluded.

### Variables analyzed

The following variables were analyzed:

- Hospital characteristics: number of inpatient beds, number of ICU beds, status as a teaching hospital, status as a trauma care referral center, existence of a transplant center, existence of a transplant coordination unit connected to the ICU, and type of hospital administration (public or private).
- Characteristics of potential donors: gender, age, Sequential Organ Failure Assessment (SOFA) score, organ dysfunctions and number of organ dysfunctions at the start of BD investigation (neurological dysfunction was not considered), cause of BD, presence of infection, and clinical management site defined as inside the ICU or outside the ICU (emergency room or postanesthesia recovery unit).
- Variables related to the checklist: compliance with the checklist was considered to indicate bedside use, regardless of the number of interventions met; compliance with each target was considered when the goal was effectively reached.
- Outcome variables: the primary outcome variable was the occurrence of CA, and the following variables related to the donation process were also evaluated: actual donations, mean number of organs retrieved per donor (ORPD), and donor loss because of family refusal or medical contraindication. The outcome variables were evaluated at 4-month intervals during the 2 years of observation and were compared with the results recorded in the previous 2 years, which served as the baseline for the indicators. The outcomes from the two 4-month periods following the observation period were also analyzed to evaluate performance consistency. Finally, to assess the impact of this improvement program in the real world, we assessed data from the annual reports of the Transplant Center of Santa Catarina between 2011 (year before the program) and 2014 (year of program completion).

### Data collection and treatment

**Fig. 1:**
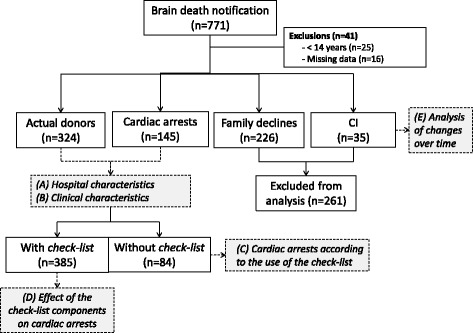
Study flowchart. *Shaded boxes* show the different analyses in the study. *CI* contraindications

The data collected were transferred to a spreadsheet for subsequent analysis. For categorical variables, inconsistencies and blank data were interpreted as protocol noncompliance or breach. Blank data or inconsistencies in the case of continuous variables were disregarded for analysis. The data were analyzed as follows (Fig. 1):

- Hospital characteristics and their relationship with CA from the donation process (Fig. 1, analysis A).
- Characteristics of PBDDs and their relationship with the outcome from the donation process (Fig. 1, analysis B).
- Compliance with the checklist and its relationship with CA (Fig. 1, analysis C).
- Relationship of each checklist component with the occurrence of CA (Fig. 1, analysis D).
- Compliance with the checklist and its association with CA over time (Fig. 1, analysis E).

### Statistical analysis

Statistical analysis was performed using Statistical Package for the Social Sciences, version 17.0 software (SPSS Inc., Chicago, IL, USA). Continuous variables were expressed as the means ± standard deviations. The Kolmogorov–Smirnov test was applied to assess the normal distribution of data. We used Student’s *t* test to compare means, and used the nonparametric Mann–Whitney test to compare the means of nonhomogeneous variances. Categorical variables were expressed as absolute and relative values and were compared using the chi-squared test. *p* < 0.05 was considered statistically significant.

The odds ratio (OR) and 95 % confidence interval (CI) were determined to evaluate the impact of checklist interventions on the loss of PBDDs from CA. The variables of interest were selected from this analysis to construct two models of multivariate prediction by logistic regression, considering the occurrence of CAs the dependent variable: a global model—hospital, potential donor, and clinical management procedure characteristics; and a checklist model—restricted to PBDDs to whom the goal checklist was applied. All variables for which a significant level of *p* < 0.10 was obtained in the univariate model were introduced into the models.

## Results

A total of 767 BD notifications occurred over six 4-month periods. Of these, 41 cases were excluded, and the other 726 PBDDs were distributed as follows: 324 (44.6 %) actual donors, 141 (19.4 %) CAs, 226 (31.1 %) family refusals, and 35 (4.8 %) contraindications (Fig. 1).

### Hospital characteristics and CA risk

The eight hospitals that received onsite training accounted for 54.9 % of the PBDDs. Hospitals with more than 250 beds, with an active organ transplant center, and with an inhospital transplant coordination unit connected to the ICU were associated with a reduced occurrence of CAs. Public hospitals and hospitals with fewer than 150 beds had a higher number of losses of PBDDs during management (Table 1).

**Table 1:** Hospital characteristics and their relationship with cardiac arrest of potential brain death donors

Characteristic	Notifying hospitals	Cardiac arrests/eligible for donation	*p* value	OR (95 % CI)
Total, *n* (%)	27 (100)	141/465 (30.3)		
Hospital beds, *n* (%)				
< 150 beds	10 (37.0)	29/62 (46.7)	0.002	2.28 (1.32; 3.93)
150–250 beds	10 (37.0)	65/217 (29.9)	0.87	
> 250 beds	7 (25.9)	47/186 (25.2)	0.053	0.66 (0.44; 1.0)
ICU beds, *n* (%)				
< 5 %	5 (18.5)	44/131 (33.5)	0.33	
5–10 %	15 (55.6)	89/294 (30.3)	0.97	
> 10 %	7 (25.9)	14/39 (35.8)	0.43	
Teaching, *n* (%)				
Yes	15 (55.6)	101/329 (30.7)	0.78	
No	12 (44.4)	40/136 (29.4)		
Trauma referral center, *n* (%)				
Yes	15 (55.6)	112/354 (31.6)	0.27	
No	12 (44.4)	29/111 (26.1)		
Transplant center, *n* (%)				
Yes	6 (22.2)	44/211 (20.8)	<0.001	0.42 (0.28; 0.65)
No	21 (78.8)	97/254 (38.1)		
Onsite training, *n* (%)				
Yes	8 (29.7)	69/238 (28.9)	0.52	
No	19 (70.3)	72/227 (31.7)		
Transplant coordination unit connected to the ICU, *n* (%)				
Yes	17 (63.0)	113/394 (18.2)	0.07	0.61 (0.36; 1.04)
No	10 (37.0)	28/70 (25.9)		
Public administration, *n* (%)				
Yes	22 (81.5)	137/433 (31.6)	0.02	3.23 (1.11; 9.41)
No	5 (18.5)	4/31 (12.3)		

For variables with more than two categories, each category was compared with the sum of the others

*OR* odds ratio, *CI* confidence interval

### Potential donor characteristics and CA risk

The clinical and demographic characteristics of the 465 PBDDs analyzed are outlined in Table 2. The odds of CA was higher among PBDDs aged > 60 years and when clinical management was performed outside the ICU, and increased in proportion to the number of organ dysfunctions observed at the start of BD investigation. The SOFA scores at the start of the BD investigation were similar between actual donors and those who experienced CA (4.9 ± 2.2 vs 5.1 ± 2.3, *p* = 0.71).

**Table 2:** Potential donor characteristics (excluding contraindications and family refusals) and their relationship with cardiac arrest

Characteristic	Total, *n* = 465 (100 %)	Cardiac arrests, *n* = 141 (30.3 %)	*p* value	OR (95 % CI)
Gender, *n* (%)				
Male	279	85 (30.5)	0.93	
Female	186	56 (30.1)		
Age group, *n* (%)				
< 20 years	38	10 (26.3)	0.57	
21–40 years	105	27 (25.7)	0.24	
41–60 years	207	58 (28.0)	0.33	
> 60 years	115	46 (40.0)	0.009	1.79 (1.15; 2.78)
Cause of BD, *n* (%)				
Traumatic brain injury	165	52 (30.9)	0.84	
Hemorrhagic stroke	161	50 (31.1)	0.80	
Ischemic stroke	56	14 (25.0)	0.35	
Subarachnoid hemorrhage	34	10 (29.4)	0.90	
Hypoxic encephalopathy	38	10 (26.3)	0.57	
Other	11	5 (45.5)	0.27	
Organ dysfunction, *n* (%)				
Cardiovascular	297	85 (28.6)	0.46	
Pulmonary	147	44 (29.9)	0.96	
Renal	120	43 (35.8)	0.13	
Hepatic	39	11 (28.2)	0.79	
Hematologic	118	34 (28.8)	0.74	
Number of organ dysfunctions at the start of BD investigation, *n* (%)				
0–1	230	55 (13.9)		1 (Reference^a^)
2	136	43 (31.6)	0.03	1.66 (1.03; 2.68)
3	70	27 (38.0)	0.004	2.26 (1.27; 4.01)
≥4	29	16 (55.2)	0.002	4.43 (1.99; 9.82)
Infection, *n* (%)				
Yes	322	96 (29.8)	0.72	
No	143	45 (31.5)		
Clinical management site, *n* (%)				
ICU	369	103 (27.9)	0.02	0.59 (0.37; 0.94)
Outside the ICU	96	38 (39.6)		

^a^We used potential donors with zero and one dysfunction as a reference to test the effect of the number of organ dysfunctions at the start of the BD investigation of cardiac arrests. For the other variables with more than two categories, each category was compared with the sum of the others

*BD* brain death, *OR* odds ratio, *CI* confidence interval

### Compliance with the checklist and its components

Table 3 shows that use of the checklist was associated with lower odds of occurrence of CA (OR 0.30, 95 % CI 0.18; 0.49, *p* < 0.001). The frequency of CAs decreased as the number of checklist items complied with increased, and the odds of those losses were substantially lower upon compliance with four or more checklist intervention items (OR 0.19, 95 % CI 0.11; 0.34, *p* < 0.001). Full compliance with the nine checklist interventions was not observed; the maximum performance levels met seven items in only nine patients.

**Table 3:** Risk of occurrence of cardiac arrest according to compliance with the checklist for potential organ donor management

Intervention and population	*N*	Cardiac arrests	OR (95 % CI)	*p* value
Without checklist	83	44 (53.0)	1 (Reference^a^)	
With checklist	382	97 (25.4)	0.30 (0.18; 0.49)	<0.001
Number of interventions, *n* (%)^a^				
1	60	21 (35.0)	0.47 (0.24; 0.94)	0.033
2	23	11 (47.8)	0.81 (0.32; 2.05)	0.65
3	62	22 (38.7)	0.48 (0.24; 0.96)	0.036
4	102	21 (20.6)	0.23 (0.12; 0.44)	<0.001
5	85	16 (18.8)	0.20 (0.10; 0.41)	<0.001
6 and 7	50	6 (12.0)	0.12 (0.04; 0.31)	<0.001
8 and 9	0	0		
Set of interventions, *n* (%)				
1–3 items	145	54 (37.2)	0.52 (0.30; 0.90)	0.02
≥ 4 items	237	43 (18.1)	0.19 (0.11; 0.34)	0.001

^a^Reference: potential donors to whom the checklist was not applied

*OR* odds ratio, *CI* confidence interval

Of 465 PBDDs, 83 were not managed with the checklist. Potential donors managed without the checklist were more frequent in the first quarter (*n* = 31, 37.5 %) and were then evenly distributed throughout the study period (10.2 ± 2.5 per quarter). Both groups (with and without checklist) were similar to each other regarding clinical and demographic characteristics and the following treatment goals: donor maintenance, duration ranging from 12 to 24 hours, temperature > 35 °C upon protocol completion, diuresis ranging from 1 to 4 ml/kg/h, serum sodium < 150 mEq/L, blood glucose < 180 mg/dl, and the number of organs donated/donor (with checklist: 2.0 ± 0.5 organs vs without checklist: 2.1 ± 0.3 organs, *p* < 0.83). In contrast, the use of corticosteroids (*p* < 0.001), vasopressin (*p* < 0.001), adequate ventilation (*p* < 0.001), and MAP levels upon management completion (73 ± 44 mmHg vs 63 ± 44 mmHg, *p* < 0.05) were higher when the management checklist was applied.

The following treatment goals were associated with reduced risk of CA among the 382 PBDDs to whom the checklist was applied: temperature ≥ 35 °C (*p* = 0.001), MAP ≥ 65 mmHg (*p* < 0.001), corticosteroid administration (*p* = 0.006), vasopressin administration to PBDDs using vasoconstrictors (*p* = 0.042), and protocol duration ranging from 12 to 24 hours (*p* = 0.07) (Table 4).

**Table 4:** Risk of occurrence of cardiac arrest among patients to whom the donor management checklist was applied according to the meeting of treatment goals

Goal met (population analyzed)	Total, *n* = 382	Cardiac arrests, *n* = 97 (25.4 %)	OR (95 % CI)	*p* value
Duration of protocol 12–24 h, *n* (%) (all)				
Yes	101 (26.4)	19 (18.1)	0.60 (0.34; 1.06)	0.07
No	281 (73.6)	78 (27.7)		
Temperature > 35 ° C, *n* (%) (all)				
Yes	283 (74.1)	60 (21.2)	0.48 (0.27; 0.74)	0.001
No	99 (25.9)	37 (37.3)		
MAP > 65 mmHg, *n* (%) (all)				
Yes	285 (74.6)	60 (21.1)	0.43 (0.26; 0.71)	<0.001
No	97 (25.4)	37 (38.1)		
Diuresis from 1 to 4 ml/kg/h, *n* (%) (all)				
Yes	210 (54.9)	49 (23.3)	0.78 (0.49; 1.24)	0.30
No	172 (45.1)	48 (27.9)		
Corticosteroids, *n* (%) (all)				
Yes	263 (68.8)	56 (21.3)	0.51 (0.32; 0.83)	0.006
No	119 (31.2)	41 (34.4)		
Vasopressin, *n* (%) (vasoconstrictor use, *n* = 236)				
Yes	41(17.4)	5 (12.2)	0.37 (0.13; 0.99)	0.042
No	195 (82.6)	53 (27.1)		
Mechanical ventilation, *n* (%) (all)				
Yes	256 (67.1)	61 (23.8)	0.78 (0.48; 1.27)	0.32
No	126 (32.9)	36 (28.6)		
Sodium < 150 mEq/L, *n* (%) (all)				
Yes	142 (37.2)	36 (25.4)	0.99 (0.61; 1.60)	0.99
No	240 (62.8)	61 (25.5)		
Blood glucose < 180 mg/dl, *n* (%) (all)				
Yes	151 (39.5)	39 (25.8)	1.03 (0.64; 1.66)	0.87
No	231 (60.5)	58 (25.1)		

*OR* odds ratio, *CI* confidence interval, *MAP* mean arterial pressure

### Multivariate analysis and the occurrence of CA

A global model of multivariate analysis was designed based on the hospital and potential donor characteristics and the clinical management goals that were associated with the occurrence of CAs (Table 5). The variables associated with decreased CAs were a hospital with more than 250 beds, performing organ transplants, management of PBDDs performed in the ICU, and use of the management checklist. Public hospitals, accounting for 81.5 % of the hospitals in the system, were associated with higher odds of occurrence of CAs.

**Table 5:** Multivariate analysis for cardiac arrest prediction in potential brain dead donors

Variable	OR (95 % CI)	*p* value
Global analysis (*n* = 465)		
Hospital characteristics		
>250 beds	0.59 (0.36; 0.98)	0.04
Organ procurement staff linked to the ICU	0.58 (0.74; 3.41)	0.23
Public hospital	9.25 (2.07; 41.1)	0.003
Active transplant program	0.36 (0.21; 0.60)	<0,001
Potential donors characteristics		
> 60 years old	1.57 (0.96; 2.57)	0.07
≥ 2 organ dysfunctions	1.48 (0.69; 2.13)	0.16
Management characteristics		
Clinical care provided inside the ICU	0.49 (0.28; 0.86)	0.013
Checklist use	0.43 (0.20; 0.62)	<0.001
Checklist analysis (*n* = 382)		
Duration of the protocol 12–24 h	0.65 (0.39; 1.15)	0.16
Temperature ≥ 35 ° C	0.68 (0.34; 1.36)	0.28
Mean arterial pressure ≥ 65 mmHg	0.54 (0.26; 1.03)	0.055
Corticoids	0.67 (0.39; 1.15)	0.15
Vasopressin	0.56 (0.32; 0.97)	0.04

*OR* odds ratio, *CI* confidence interval

Based on the findings presented in Table 4 we performed a multivariate analysis model restricted to the 382 PBDDs to whom the checklist was applied to assess the individual effects of checklist components on CAs (Table 5). The variables best associated with decreased CAs were the administration of vasopressin for donors using vasoconstrictors and MAP ≥65 mmHg upon protocol completion.

### Compliance with the checklist and its association with CAs and ORPD over time

**Fig. 2:**
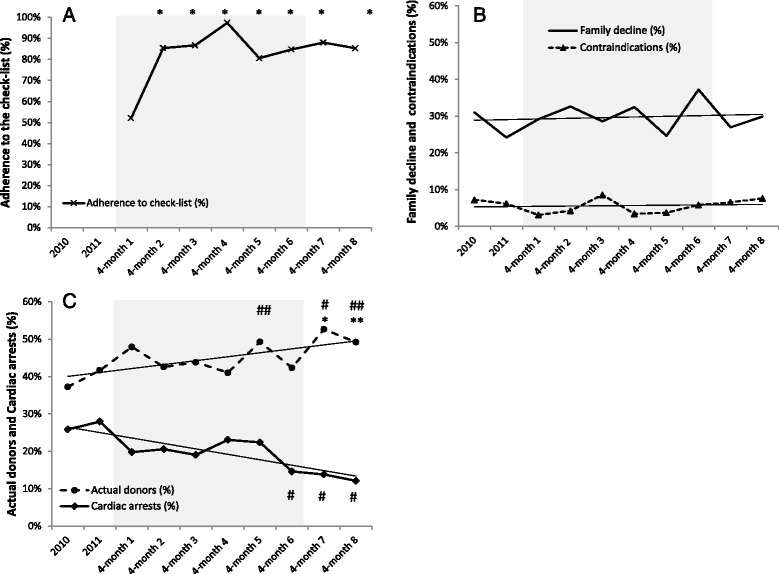
Changes in (**a**) adherence to checklist (%), (**b**) family decline (%) and contraindications (%), and (**c**) CAs (%) and actual donors (%) over time. *Shaded area* refers to 2 years of data collection divided into 4-month periods. *White Areas* refer to the previous 2 years and the two subsequent quarters of the observation period. *p < 0.01 and **p < 0.05 related to first 4-month period. ^#^ *p* < 0.01 and ^##^ *p* < 0.05 compared with previous 2 years of the study period

Increased compliance with the checklist occurred between the first and second 4-month periods (52.1 % vs 85.8 %, *p* < 0.001), with rates around 80 % maintained until the end of the study (Fig. 2a).

Family refusals and contraindications remained constant throughout the intervention period (Fig. 2b). The comparison between the two study years and the performance of the state transplant system in the two previous years (CA: 27.3 %) showed downward trends in CAs in the second (20.6 %, *p* = 0.08) and third (19 %, *p* = 0.09) 4-month periods. After 24 months, CAs decreased to 14.6 % (*p* = 0.002), reaching 12.1 % in the following two 4-month periods (*p* < 0.001). Simultaneously, increases in actual donations were observed (Fig. 2c). At the baseline, the mean number of ORPD was 2.54 ± 1.08, which increased to 2.78 ± 1.03 ORPD (*p* = 0.11) after 24 months and to 2.90 ± 0.93 ORPD (*p* < 0.004) in the following two 4-month periods.

The analysis of the annual records of the Transplant Center of Santa Catarina showed the following rates for 2011 and 2014 respectively: BD notifications, 61.5 per million population (pmp) and 75.1 % (*p* = 0.003); family refusal, 37 % and 38.5 % (*p* = 0.21); clinical contraindications, 5.2 % and 11.9 % (*p* < 0.001); CAs, 27.1 % and 13 % (*p* < 0.001); and effective donations, 25.4 pmp and 32.3 pmp (*p* = 0.02). The mean number of ORPD also increased in this period (2011: 2.61 ± 0.82; 2014: 2.99 ± 0.96, *p* < 0.001).

## Discussion

Using the VIP approach checklist reduced the odds of CA in PBDDs prior to organ harvesting 2.3-fold, increasing actual multiple organ donations over time. The number of CAs was inversely proportional to the number of treatment goals met. Hence, the VIP approach seems to be useful to increase awareness of physicians to guarantee a “standard of care” during the management of PBDDs. To our knowledge, this is the first multicenter report providing evidence of benefits from a management protocol of potential organ donors guided by a goal checklist in a developing country.

This program for improvement of BD donor management had the characteristics of a task force for dissemination and application of evidence-based clinical practice guidelines of PBDD management endorsed by the national associations of critical care and transplantation [5, 6]. The main recommendations from the Guidelines were organized into a goal checklist used to guide case management. Our local experience with a management protocol enabled establishing a strong association between attaining a set of goals organized into bundles and decreasing CAs among PBDDs [14]. The prospect of broadening the results from that pilot study prompted this regionwide program to improve the management of PBDDs. Inspired by the Surviving Sepsis Campaign, this donor management optimization initiative consisted of an institutional process of performance improvement which, according to its own characteristics, does not meet all scientific rigor required to assess the impact of the best evidence on clinical outcomes [10].

From our perspective, the VIP approach checklist is an alternative to the existing donor optimization care bundles, proposing an adaptation of a classic strategy for the management of the circulatory shock [16]. The VIP approach provides a simple sequence of procedures intrinsically directed to the restoration of DO_2_ and shock control, which is present in 80 % of PBDDs and is the leading cause of CA in these individuals [15].

Despite the positive impact of our checklist on CA, a rate of 12.1 % of CAs may sound high for some. In this context, it is important to consider that the incidence of CA reported in our results accounts for all events that occurred after starting the first BD diagnostic test, which will result in a necessarily larger number of CAs than those presented in countries which take into account only the CAs that occurred in consented PBDDs.

The annual records of the Transplant Center show that the reduction of losses of donors by CA (2011: 27.1 % to 2014: 13 %, *p* = 0.002) strongly contributes to increasing the effective donations in the state of Santa Catarina (32.3 pmp), which is already close to rates reported by the best world performances of Spain and Croatia (35 pmp), and even higher than Portugal (28.3 pmp) or the United States (25.9 pmp) [18]. Moreover, it was possible to observe an additional effect of the clinical optimization provided by the VIP approach, which not only reduced the number of unexpected CAs, but also increased the suitability of organs.

Our results are consistent with some reports presented earlier. Salim et al. [1] showed that an aggressive strategy of deceased potential donor management reduced the loss of organ donors due to CA by 87 % (*p* < 0.001) and increased the number of organ donors by 82 % (*p* < 0.001) over 8 years. A series of publications from the Organ Procurement Organizations (OPOs) of the United Network for Organ Sharing (UNOS) has shown that standardization of procedures of PBDD management towards meeting clinical goals can increase the number of donors and the number of organs transplanted per donor [9–14, 19, 20].

The fact that public hospitals had an increased association with the risk of CAs may be partly explained by infrastructure problems, including the availability of ICU beds. There was a high number of PBDDs (20.6 %) managed outside the ICU, where the treatment conditions may be suboptimal. Although our study was conducted in one of the most developed regions of the country, with a number of ICU beds similar to that of USA and many European countries, we have to consider the local heterogeneity of health care resources. There are differences in infrastructure between public and private hospitals and even among public hospitals, depending on the degree of complexity of each institution [21, 22]. Furthermore, donor losses due to CA in public institutions were mitigated in hospitals with more than 250 beds and in those performing transplantations (Table 5), which are also indicative of better infrastructure and organizational conditions.

The odds of CA have been higher among PBDDs > 60 years old and those with more organ dysfunction at baseline. However, these variables did not stand out as CA predictors in the multivariate analysis. Considering the nearly absolute vasopressin deficiency that occurs in the first minutes after BD [23, 24], early replacement of this hormone has been formally recommended for the 80 % of PBDDs needing vasopressors [5, 6, 25–27]. Vasopressin replacement is independently associated with an increased rate of organ recovery and a less overall graft rejection due to poor function [28]. The good pressure control related to vasopressin administration [24] corroborates the association of vasopressin use and obtaining MAP ≥ 65 mmHg with the decrease in CAs observed in our results. However, meeting isolated goals such as MAP ≥ 65 mmHg may not suffice to prevent CA among PBDDs [10]. This point is reinforced by the observation that the decrease in the number of CAs was more consistent when a greater number of interventions were used in combination (Table 3), reflecting the quality of care provided.

Despite the good compliance with the checklist already observed in the first months, donor loss due to CA decreased slowly over time (Fig. 2). The prioritization of hospitals with a history of a high number of organ donors, the slower recruitment of lower-performance hospitals, and the learning curve of care teams may explain this delay. Additionally, even with an active education program, the incorporation of new evidence and guideline recommendations may take years and usually fail to achieve rapid impacts at the bedside.

It could be seen as negative that a checklist is needed to do what should be done, but it is known that health care centralized on the physician may result in noncompliance with guideline recommendations, especially in highly complex processes such as the management of donors [29]. In contrast, there are many demonstrations that the systematic use of multidisciplinary checklists as alert devices for the physician improves the process of care, decreasing catheter-related bloodstream infections [30, 31], surgical-related morbidity and mortality [32], and clinical outcomes in the ICU [33]. It is also known that the mere existence of a checklist does not ensure its effective application, highlighting the importance of managing the bedside protocol, wherein a case manager advises the care team [34]. In the context of PBDD management, inhospital transplantation coordinators should be encouraged to play the leading role of case managers, alerting the care team based on a goal checklist.

Our study has some limitations. First, the observational nature of this study only allows us to infer associations between the use of the checklist and the decrease in the loss of donors due to CA. Second, the data on therapeutic interventions performed on PBDDs to whom the checklist was not applied are not entirely reliable because these data were derived from secondary records. Third, we did not evaluate the quality of the organs donated or graft function and survival, which limited the scope of the analysis of the effects of interventions. Fourth, regional factors that may influence the total time of the PBDD maintenance process and therefore the occurrence of CA in PBDDs must be considered, such as the differences in BD diagnosis methodologies.

The overlap of medical, administrative, logistical, and legal aspects during the donation process makes the management of PBDDs extremely complex, sometimes stressful, and highly dependent on the involvement of an organized care team. The use of clinical protocols in this situation may help as a guidance and alert tool towards meeting goals. To promote adherence to clinical protocols, three fundamental aspects must be followed: to set goals based on the best available evidence; to standardize management; and to simplify the protocol to the maximum. In addition, standards of care provide opportunities for future observational studies or randomized trials that can help to provide new and better evidence on this subject.

## Conclusions

A quality improvement program based on education and the use of the VIP approach for the bedside management of PBDDs is strongly associated with a reduction in donor loss from CA, increasing actual donors and ORPD. Organ transplant hospitals, clinical management performed in the ICU, obtaining MAP ≥ 65 mmHg, and vasopressin use are factors that also mitigate the occurrence of CAs. Such a checklist may promote staff commitment to the quality of care during the management of PBDDs.

## Key messages

- The management of PBDDs is extremely complex and depends on the involvement of a highly organized care team.
- The systematic application of a checklist for the clinical management of PBDDs is useful to increase physicians’ awareness and the quality of care.
- The use of clinical protocols during PBDD management may help the care team as a guidance and alert tool towards meeting goals.
- Meeting clinical goals during clinical management of PBDDs leads to a progressive reduction in CAs, contributing to an increase in actual donors.
- Governmental and/or associative initiatives promoting coordinated actions of the donation and transplant process may amplify the effects and strongly contribute to reducing the imbalance between the supply and demand of transplant organs.

## Additional file

Additional file 1: is **Table S1** presenting the sequence of diagnostic and therapeutic measures applied to the potential organ donor according to the VIP approach. (DOCX 17 kb)

## Abbreviations

BD: Brain death
CA: Cardiac arrest
CI: Confidence interval
DO_2_: Oxygen delivery
FiO_2_: Fraction of inspired oxygen
MAP: Mean arterial pressure
OPO: Organ Procurement Organization
OR: Odds ratio
ORPD: Organs retrieved per donor
PBDD: Potential brain-dead donor
PEEP: Positive end-expiratory pressure
pmp: Per million population
SaO_2_: Arterial oxygen saturation
SOFA: Sequential Organ Failure Assessment
UNOS: United Network for Organ Sharing
VIP: Ventilation, Infusion and Pumping
VIPPS: Ventilation, Infusion, Pumping, Pharmacological support and Specificities
