# Supplementary material for: Positive impact of a clinical goal-directed protocol on reducing cardiac arrests during potential brain-dead donor maintenance
Source: Crit Care. 2016 Oct 11;20:323. doi: 10.1186/s13054-016-1484-1 (PMC5057215; doi:10.1186/s13054-016-1484-1)
Supplement: Additional file 1: — is Table S1 presenting the sequence of diagnostic and therapeutic measures applied to the potential organ donor according to the VIP approach. (DOCX 17 kb) [file 13054_2016_1484_MOESM1_ESM.docx]

**Table S1.** Sequence of diagnostic and therapeutic measures according to the *VIP approach* used as the basis for the management checklist*.*

| **Priority** | **Treatment** | **Goals** |
| --- | --- | --- |
| 1 | V_entilation_ | **Protective ventilation**  - Normal lungs: Vt = 6 to 8 ml/kg (ideal body weight)  PEEP = 8-10 cm H_2_O and FiO_2_ to obtain SaO_2_ > 90%  - ARDS: Vt = 6 ml/kg (ideal body weight)  PEEP and FiO_2_ titration to obtain SaO_2_ > 90% |
| 2 | I_nfusion_ | **Initial volume expansion**  - If MAP < 65 mm Hg or diuresis < 1 mL/kg/h, infuse 30 mL/kg of crystalloid  (Use CVP or ∆Pp to guide additional infusion)  **Eletrolytes** (Goal: Na^+^ < 155 mmol/L and normal values of K^+^, Mg^++^, PO4^-^,Ca^++^)  **Blood products** (Goal: Hb 7 to 10 g/d) |
| 3 | P_ump_ | **Fluid responsiveness evaluation after initial volume expansion**  - Infuse more fluid only if CVP < 5 mm Hg or ∆Pp ≥ 13%  **Temperature** (Goal: Core temperature arround 35^o^C)  - If severe hemodynamic instability, core temperature above 35^o^C may be safer  **Cardiac arrhythmias** (Goal: sinusal rhythm and HR between 60-120 bpm)  ATTENTION: Bradyarrythmias are unresponsive to atropine in brain death. |
| 4 | P_harmacological_ ^treatment of shock^ | **If MAP < 65 mm Hg despite initial infusion of 30 mL of crystalloid**  - Noradrenaline or dopamine plus vasopressin 1 U + 0,5-2,4 U/h  - Dobutamine only when there is evidence of ventricular dysfunction |
| 5 | S_pecific_ ^management of the etiologic cause of shock^ | **Hormone replacement** (Goal: MAP ≥ 65 mm Hg and diuresis < 4 mL/kg/h)  - Vasopressin when administering noradrenaline/dopamine/adrenaline  Polyuria control in *diabetes insipidus*  - Vasopressin 1 U + 0,5-2,4 U/h (in donors requiring vasoconstrictors)  - Desmopressin 1-2 µg 4/4 h (in cases that vasocontrictors are not required)  - Triiodothyronine (T_3_)*  - Methylprednisolone* 15 mg/kg/24 h or hydrocortisone* 50 mg 6/6 h  - Continuous infusion of insulin for hyperglicemic donors (Goal: < 180 mg/dL)  **Sepsis -** cultures and appropriate broad antibiotic therapy should be performed |

*Indication to be made in accordance of a local policy or solicitation of the transplant team.

CVP: central venous pressure, ∆Pp: pulse pressure variation, FiO_2_: fraction of inspired oxygen, Hb: hemoglobin, HR: heart rate, MAP: mean arterial pressure, PEEP: positive end expiratory pressure. Vt: tidal volume.

**Original source:** Westphal GA. A simple bedside approach to therapeutic goals achievement during the management of deceased organ donors – An adapted version of the “VIP” approach. [Clin Transplant.](http://www.ncbi.nlm.nih.gov/pubmed/26588881) 2016 Feb;30(2):138-44
